# Patterns of Women’s Postpartum Weight Retention and Its Associations with Maternal Obesity-Related Factors and Parity

**DOI:** 10.3390/ijerph16224510

**Published:** 2019-11-15

**Authors:** Tingting Sha, Gang Cheng, Chao Li, Xiao Gao, Ling Li, Cheng Chen, Yan Yan

**Affiliations:** Department of Epidemiology and Medical Statistics, Xiangya School of Public Health, Central South University, Xiangya Road 110, Changsha 410078, China; tingtingsha@csu.edu.cn (T.S.); gangcheng@csu.edu.cn (G.C.); JenniferLiChao@163.com (C.L.); 18670321975@163.com (X.G.); 18674824070@163.com (L.L.); chengchen_8493@126.com (C.C.)

**Keywords:** body mass index, parity, postpartum weight retention, waist circumference, weight gain

## Abstract

Background: There is not much data on the effects of the timing of gestational weight gain (GWG), pre-pregnancy waist circumference (WC), pre-pregnancy body mass index (BMI), and parity, with postpartum weight retention (PPWR) trajectories. Methods: This study was based on a longitudinal cohort. Latent growth mixture models were applied to identify the latent trajectories of PPWR and test the effects of the predictors on distinct classes of PPWR trajectories. Results: Three PPWR trajectories were identified. About 2.8% (*n* = 26) of women were classified into Class 1, with an inverted U-shape trajectory; 6.6% (*n* = 61) were assigned to Class 2, with a rapid increase trajectory; 90.6% (*n* = 837) were classified into Class 3, with a significant decrease. Women who had a lower pre-pregnancy BMI (β = −0.279), higher pre-pregnancy WC (β = 0.111) and GWG (β = 0.723) were at a higher risk of retaining more weight at 1 month postpartum. Only GWG, especially GWG during late pregnancy, was associated with the rate of PPWR change. Parity was not associated with the changes in PPWR, while, compared to Class 1 trajectory, multiparous women were protected from having a Class 2 trajectory. Conclusions: Early targeted interventions should be taken to prevent women who were primiparous, and/or had a lower pre-pregnancy BMI and higher pre-pregnancy WC and GWG, from excessive PPWR.

## 1. Introduction

Pregnancy is one of the biological and natural processes which can trigger physiological changes in body composition and cause significant weight gain in childbearing-aged women [1]. Childbearing-aged women are vulnerable to excess weight gain and weight retention during the first year after delivery [2]. Compared with weight gain during other periods of life, excess weight gain retained after childbirth seems to be more harmful, as evidence suggests that it is more likely to be central fat deposition [3]. Although many women try to return to their pre-pregnancy weight after giving birth [4], approximately 75% of women failed to return to their pre-pregnancy weight 1 year postpartum [5], and a number of these ultimately become obese and overweight [6]. Data reported by previous studies indicate that the average weight retained was about 1.56–4.1 kg at 6 months postpartum [7] and 0.5–1 kg in the first year of postpartum [8], with substantial variability in PPWR, from a gain of 26.5 kg to a loss of 12.3 kg [9,10].

Emerging evidence demonstrated that excessive PPWR during childbearing years is associated with the development of adverse maternal health outcomes, such as cardiovascular diseases, metabolic syndromes, and long-term obesity in later life [11]. Thus, it is critical to prevent excessive PPWR during the pregnancy–postpartum period. Factors related to PPWR have been extensively identified, such as GWG [12,13], pre-pregnancy BMI [14,15], parity [16,17], breastfeeding practices [18], and a lack of physical exercise. However, except for GWG, no consistent conclusions were found for other factors, and several gaps in the evidence base challenge the existing results [7].

First, previous research documented a significant correlation between pre-pregnancy BMI and PPWR, while the role of maternal pre-pregnancy BMI in the rate of the growth and development of maternal PPWR changes is less clear and incompletely studied. Second, when assessing the effect of GWG on PPWR, researchers usually focus on total GWG. Weight gain across specific intervals of pregnancy may contribute differently to maternal outcomes [19,20,21]. For example, Gonzalez et al. found that GWG in the third trimester is a determinant of pregnancy-induced hypertension, while it has a negative relationship with gestational diabetes mellitus [22]. The GWG rate during 22–26 weeks was consistently associated with an elevated diastolic blood pressure level [23]. Thus, researchers should pay more attention to the implication of timing of weight gain in pregnancy with PPWR, which might help to reduce excessive postpartum weight during the pregnancy period [24]. Third, numerous studies demonstrated that a higher pre-pregnancy waist circumference (WC) can reflect more visceral fat and central adiposity, and is also considered a better predictor of obesity-related diseases than BMI [25,26]. However, its role in maternal PPWR remains unclear [7]. Fourth, the association between parity and PPWR has yielded conflicting findings in previous studies, with several [27,28], but not all [29,30,31], suggesting that parity may be a proxy for the number of times a woman has struggled with excessive GWG or PPWR.

Lastly, the trajectory of PPWR is not the same for all women. Research estimating different classes of PPWR growth trajectories over time is rare, which would mask group differences in weight retention trajectories. Therefore, determining the patterns of PPWR changes for different populations, and examining the potential risk factors for different trajectories, is beneficial to discover individuals who are susceptible to excess PPWR. The current study aimed to describe the patterns of PPWR for different samples based on a Chinese longitudinal cohort, and investigate the effects of maternal parity, timing of GWG, pre-pregnancy WC, and BMI on PPWR trajectories.

## 2. Materials and Methods 

### 2.1. Study Population

Changsha has completed maternal and child health care records in the Community Health Management Information System (CHMIS). Considering its long-term cooperation with the Health Bureau of Kaifu District, we selected three Community Health Service Centres (CHSCs) of Sifangping, Dongfenglu, Xinhe streets of Kaifu District of Changsha to ensure the project was carried out successfully. 

The baseline survey was conducted within 15 days after delivery from January 1, 2015, to December 31, 2015, and the follow-up surveys were conducted at 1, 3, 6, and 8 months postpartum. The participants included in the baseline survey were restricted to respondents who were permanent residents in Kaifu District, had delivered live-born babies, provided completed health care records in the CHMIS, had no history of mental illness or brain disease, and gave written informed consent. Overall, 976 eligible mothers participated in the cohort. A total of 52 mothers were excluded for the following reasons: 45 of the mothers lacked the data on antenatal care information or initiated antenatal care later than 12 weeks of gestation, and seven of the mothers were excluded due to missing predictors, such as pre-pregnancy WC. Finally, 924 of the mothers were included in the final data analysis.

### 2.2. Assessment for Obesity-Related Factors and Parity

Maternal weight and height during the pregnancy were measured by trained nurses at the CHSCs. Weight was measured within the first trimester as a proxy for pre-pregnancy weight. Previous studies have demonstrated good validity and a strong correlation between the first measured weight in first-trimester pregnancy and pre-pregnancy weight (0.95, *p* < 0.01) [32,33]. Pre-pregnancy BMI was categorized into four groups according to the Working Group on Obesity in China [34]: underweight (BMI < 18.5 kg/m^2^), normal weight (18.5 kg/m^2^ ≤ BMI < 24.0 kg/m^2^), overweight (24.0 kg/m^2^ ≤ BMI < 28.0 kg/m^2^) and obesity (BMI ≥ 28.0 kg/m^2^). GWG was calculated as the difference between pre-pregnancy weight and maternal delivery weight. On the basis of pre-pregnancy BMI, GWG was categorized into three categories: inadequate, adequate, and excessive, as recommended by the 2009 Institute of Medicine (IOM) [35]. The IOM-recommended proper weight gain is 12.5–18 kg for underweight women; 11.5–16 kg for normal-weight women; 7–11.5 kg for overweight women and 5–9 kg for obese women. Early GWG was calculated as the difference between the first measured weight, measured within 12 weeks, and the weight recorded between 24 and 27 weeks. Later GWG was calculated as the difference in weights measured at weeks 24–27 and 32–36 [17]. Very late GWG was calculated as the difference in weights measured at weeks 32–36 and the delivery weight. Pre-pregnancy WC was measured at the first prenatal care and was classified by Chinese standard: <80 cm for non-central adiposity and ≥80 cm for central adiposity [25]. Parity was based on recorded data from the CHMIS and was categorized into two categories, primiparous and multiparous.

### 2.3. Assessment for PPWR

Maternal postpartum weight was mainly collected using the electronic weighing scale in their residences, while a few mothers’ postpartum weight was collected based on their self-report at 1, 3, 6, and 8 months after delivery. PPWR was calculated as the difference between the self-reported postpartum weight and weight before pregnancy at each time point. Previous studies demonstrated that self-reported weight was sufficiently accurate and reliable [36,37].

### 2.4. Data Collection

The self-designed questionnaire and maternal health care records in CHSCs were used to collect relevant information through face-to-face interviews at the baseline survey and 1, 3, 6, and 8 months postpartum. The following covariates that might have effects on PPWR were identified and assessed: maternal age, maternal education attainment, household income, gestational week, delivery mode, maternal parity, pregnancy-related complications (such as gestational diabetes mellitus, pregnancy-induced hypertension, etc.), passive smoke during pregnancy, physical exercise, depressive symptoms and mode of feeding. Data on maternal age, gestational week, delivery mode, parity, pregnancy-related complications were collected based on the maternal health care records in CHSCs at the baseline survey within 15 days after delivery. Data on maternal education attainment, household income, mode of feeding, and maternal depressive symptoms were collected by asking the mothers at 1 month postpartum through face-to-face interviews. Maternal depressive symptoms were assessed at 1 month postpartum, using the Edinburgh Postnatal Depression Scale (EPDS), with a total score ranging from 0 to 30.

### 2.5. Statistical Analysis

Continuous variables are presented as mean and standard deviation if normally distributed, and categorical variables are presented as numbers and proportions. T-tests were applied to compare the differences in PPWR by dichotomous variables, such as waist circumference, parity, depression, feeding mode, delivery mode, and passive smoke during pregnancy. One-way analysis of variance analyses were used to compare the PPWR differences within polytomous variables, such as maternal education level, household income, pre-pregnancy BMI group, and GWG group.

To model the heterogeneity in the subpopulations, we applied the latent growth mixture model (LGMM) to identify the patterns of PPWR change overtime. LGMM was a method for identifying multiple unobserved subpopulations, describing the longitudinal change, and examining differences in change among latent subpopulations [38]. The PPWR trajectory across time was modeled with continuous latent variables; latent intercept growth factor, representing the initial level of the weight retention; and latent slope growth factor, reflecting the rate of the PPWR change. The distinct subpopulations were modeled using the categorical latent variables (classes).

Three steps of the data analysis were employed to identify the latent classes of PPWR and investigate the predictors of the latent trajectories. In the first step, we modeled the trajectory of PPWR in three assumptions (for linear, quadratic, or freely estimated) to determine the most suitable model. The second step of the model specification was to identify the appropriate number of classes that yielded the most desirable fit. We compared two- to seven-class unconditional LGMM models (with no covariates). The best-fitting LGMM of the PPWR trajectories had the lowest information criteria and high entropy for the confidence of classification [39]. In the final stage, we extended the unconditional LGMM to a conditional LGMM with predictors. Considering that too many covariates may impede model convergence, we only included the covariates which were first suggested by univariate analyses. Finally, in post hoc analyses, we tested the effects of predictors on the identified latent classes of PPWR. 

A variety of model-fit indices were used to evaluate the goodness of LGMM: χ2 statistic, likelihood ratio chi-square, Akaike Information Criterion (AIC), Bayesian Information Criterion (BIC), and sample size-adjusted BIC and entropy. A higher value of entropy indicated less classification error, and if the value was >0.8, it suggested that the model had an adequate classification quality [40]. Lo-Mendell-Rubin adjusted likelihood ratio test (LMR-test) was used to compare n-class model versus n-1-class models. The significant p-value suggested that the n-class model was more suitable than the n-1-class model [41]. The models were estimated using the robust maximum likelihood approach. One hypothesis in this research was that data were missing at random. Missing data were modeled using a full-information maximum-likelihood method, which estimates the parameters based on all available data [42]. 

The descriptive statistics were conducted using SAS version 9.4 (SAS Institute, Cary, NC, USA). LGMM analyses were performed using MPLUS version 7.0 (Muthén and Muthén, Los Angeles, CA, USA). All tests were performed at the level of significance of α = 0.05.

### 2.6. Ethics Approval and Consent to Participate

This study was conducted under the approval of the independent Ethics Committee (EC) of the clinical pharmacology Institute of Central South University (CTXY-130041-3-2).

## 3. Results

Table 1 shows the socio-demographic characteristics of the participants. The mean maternal age and gestational weeks at delivery were 29.90 ± 3.91 years and 38.93 ± 1.51 weeks, respectively. The mean pre-pregnancy WC was 75.64 ± 5.21 cm, with 17.7% of mothers having central adiposity. The mean pre-pregnancy BMI was 21.25 ± 3.01 kg/m^2^, with 65.9% mothers having normal weight. About 18.5% of mothers were underweight, and 15% overweight or obese. According to the recommendations by the Institute of Medicine, 20.6% of participants had inadequate weight gain, and 33.0% gained more than the recommended weight gain. 

Changes in PPWR over time by predictors and covariates are presented in Table 2. The results of the one-way repeated analysis of variance show that pre-pregnancy WC, pre-pregnancy BMI, and GWG were significantly associated with the PPWR over time. Parity, mode of delivery, and smoking status during pregnancy were correlated to the PPWR at 1 month postpartum, while other covariates were not associated with the PPWR. Table S1 shows the results of the comparison of covariates by different parity, pre-pregnancy BMI, pre-pregnancy WC, and GWG groups.

For the first step, we modeled three latent growth mixture model (LGMM) parameter estimates for the trajectories of maternal PPWR to identify the most appropriate factor loadings. Fit indices for the three unconditioned LGMMs are summarized in Table 3. The results show the quadratic model provided a superior enhancement in model fit than the other two models, with the lowest LL-value, Akaike information criterion (AIC) value, Bayesian information criterion (BIC) value, BIC value, and the highest Entropy. Therefore, the quadratic model was used to describe the trajectory of PPWR across time. 

The possible six trajectories models with the goodness of fit indices are presented in Table 4. After comparing the fitness statistics comprehensively, the three-class model was identified as the optimal fitting model representing weight retention changes. 

**Figure 1:**
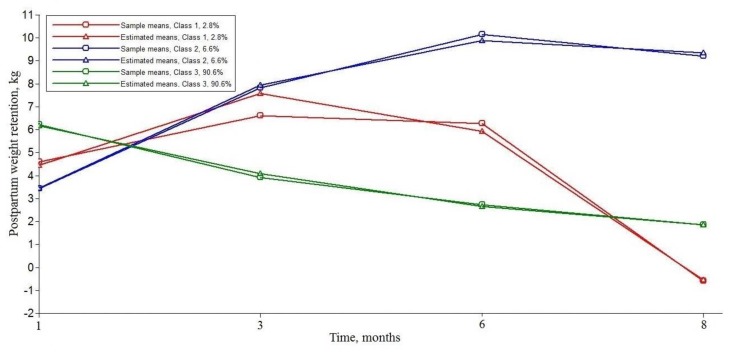
Three distinct latent trajectories of postpartum weight retention.

The distinct PPWR trajectories for the three-class solution is presented in Figure 1. Class 1 trajectory (*n* = 26, 2.8%) showed an inverted U-shape trajectory which started at a moderate PPWR at 1 month postpartum. Approximately 6.6% of the sample (*n* = 61) was assigned to Class 2 trajectory, with the lowest maternal weight retention at 1 month postpartum and a rapid increase in the rate of PPWR during the first eight months after delivery. More than 90% of participants were classified into Class 3 trajectory (*n* = 837), with a higher initial level of weight retention and a significant decrease in the rate of PPWR. The overall trajectory of PPWR is shown in Figure S1. As more than 90% of participants were classified into Class 3, the changes in the overall trajectory for PPWR were mostly close to the Class 3 trajectory. 

Table 5 shows the estimates for the three-class conditioned quadratic model. Pre-pregnancy BMI was negatively associated with the initial level of weight retention (β = −0.28, 95% confidence interval [95% CI], −0.40, −0.16), while higher pre-pregnancy WC and GWG were significantly associated with the increased baseline PPWR (β = 0.11, 95%CI, 0.04 to 0.19 and β = 0.72, 95% CI, 0.66, 0.78). Additionally, only GWG had effects both on the slope (β = −0.26, 95% CI, −0.33 to −0.19) and quadratic slope (β = 0.04, 95% CI, 0.02 to 0.06) of the PPWR change. In this study, no significant association was found between the baseline PPWR, the rate of PPWR changes and maternal parity. Additionally, the result showed a significant negative association between delivery mode and baseline PPWR, suggesting that cesarean delivery might contribute to higher initial levels of weight retention. Our result showed that exclusive breastfeeding at 1 month postpartum was associated with a lower PPWR. Furthermore, we also examined the effects of early GWG, late GWG, and very late GWG on the trajectory of PPWR, respectively. We found that the early GWG and late GWG were both related to initial levels of PPWR (β = 0.51 and 0.52, *p* < 0.001) and the slope of the PPWR (β = −0.16 and −0.25, *p* < 0.001), while only GWG during late pregnancy contributed to the quadratic slope (β = 0.04, *p* < 0.001) of the trajectory of PPWR (Tables S2 and S3). GWG during the very late pregnancy was only associated with initial levels of PPWR (Table S4). 

Comparisons of the predictors of a three-class trajectory based on the post hoc logistic regressions are presented in Table 6. Considering the PPWR trajectory, which was close to the pre-pregnancy weight at 8 months postpartum, was the main focus of our concern, we selected Class 3 as the referent class trajectory and used the logistic regressions to investigate the associations of interest predictors among Class 1, 2 and 3 trajectories. Compared to the general PPWR Class 3 trajectory, a higher pre-pregnancy BMI was strongly correlated to the increased probability of membership in the Class 1 trajectory (Odds ratio [OR] = 1.28, *p* < 0.001), and Class 2 trajectory (OR = 1.41, *p* < 0.001). In comparison to the Class 3 trajectory, a higher GWG was strongly related to the increased probability of membership in Class 2 trajectory (OR = 1.11, *p* < 0.01). Multiparous was associated with a lower PPWR in the Class 2 trajectory in comparison to the Class 3 trajectory (OR = 0.50, *p* < 0.05).

## 4. Discussion

This study explored the 8-month postpartum trajectories for PPWR in a Chinese community-dwelling population. Using LGMM, three latent class trajectories were identified. Our findings suggested that women who had a lower pre-pregnancy BMI, higher pre-pregnancy WC and higher GWG, especially a higher GWG during late pregnancy, were associated with increased initial levels of weight retention. Additionally, we found that only higher GWG contributed to the rate of PPWR changes. Parity played no role in the baseline and the rate of PPWR, while, compared to the Class 1 trajectory, multiparous women were protected from having a Class 2 trajectory. Importantly, the results also demonstrated that obesity-related risk factors played different roles in distinct latent trajectories of the PPWR.

Consistent with our results, previous studies indicated that women retained about 1–5.5 kg at 6–12 months postpartum [43]. A recent study also identified three trajectories of postpartum weight, with most of the participants having lower weight retention initially and slight weight gain over 3 years [18]. Our findings suggested that the trajectory of PPWR can be well-depicted in a quadratic LGMM, with substantial variability in postpartum weight development—both in the initial status and the rate of weight retention overtime. Most women’s weight (Class 3) had a slight decrease in eight months and finally became close to the pre-pregnancy weight. About 6.6% of women (Class 2) had a lower level of weight retention at 1 month postpartum, and a rapid increase in PPWR during the first eight months after delivery, where their weight was significantly higher than their pre-pregnancy weight. In Class 1 (2.8%), women showed an obvious decrease in PPWR from 3 months postpartum and had a lower weight than their pre-conception weight at 8 months postpartum. Compared to the reference Class 3 trajectory, we can easily find that maternal obesity-related factors and parity played different roles in Class 1 and 2 PPWR trajectories. Gaining insight into the association between potential factors and PPWR would enable the development of more targeted behavior-change interventions.

After adjusting for the covariates, our results suggested that a lower pre-pregnancy BMI was associated with a higher PPWR, which was consistent with a recent meta-analysis including 10 studies, which reported a linear association between pre-pregnancy BMI and PPWR [14]. They showed that, in comparison to the normal-weight women, women with obesity or overweight retained less weight, while women who were underweight retained more weight. A Chinese study found that overweight women retained less weight than normal-weight and underweight women [44]. 

Similarly to previous studies [45,46,47], our findings indicated that GWG was the most important predictor of PPWR. In the present study, GWG was the only factor that was associated with both the initial levels of weight retention and the rate of PPWR changes, indicating a key to reducing PPWR [48]. Furthermore, we found that, compared to the GWG during early and very late pregnancy, GWG during late pregnancy contributed more to the baseline PPWR and to the rate of PPWR changes, which is consistent with the previous Chinese study [49]. They found that Chinese women who gained more weight during the second and third trimesters retained more weight at 6 weeks and 3 months postpartum. This effect was seldom examined by previous studies and can be interpreted as second trimester weight gain in women predicting most of the excessive GWG in pregnancy [50]. These findings are essential, as identification of women who gained excess weight in the second trimester of pregnancy may help customize prenatal care and nutritional counseling in late pregnancy, to prevent greater PPWR. 

The association between parity and PPWR varied from other researches. Some studies showed that multiparous women retained less weight at six months postpartum [51], while other studies found that weight retention was higher in multiparous women [52,53]. The present study showed that primiparous women tend to retain more weight than multiparous women at 1 month postpartum. However, this association turned non-significant after controlling for covariates. Compared to the Class 1 trajectory, whose postpartum weight was mostly close to pre-pregnancy weight, primiparous women were associated with the Class 2 trajectory, showing a significant increase in PPWR. A recent meta-analysis suggested that parity was significantly associated with higher pre-pregnancy BMI, but its role in GWG and maternal PPWR remains unclear [54]. They assumed that it is likely that the influence of parity on PPWR is indirect and complex. 

Additionally, the present study showed that a higher pre-pregnancy WC was a positive predictor for higher PPWR. Although limited studies investigated the influence of pre-pregnancy WC on maternal PPWR, previous research has suggested that WC is more sensitive and more effective in evaluating cardiovascular disease risks in women, such as type 2 diabetes and cardiovascular diseases, as well as adverse pregnancy outcomes such as GDM [55,56]. Higher weight retention, as a comorbidity of obesity-related adverse pregnancy outcomes, might be well predicted by the pre-pregnancy WC. Further studies should be conducted to provide more evidence on this association to support our result.

Previous research estimating different classes of PPWR growth trajectories over time is rare, which will mask group differences in weight retention trajectories. From our results, we find that these predictors played different roles in different latent trajectories of PPWR. For example, compared with women who were mostly close to their pre-conception weight (Class 3), primiparous women, along with women had a cesarean delivery, higher pre-pregnancy BMI or excessive weight gain, were more likely to experience the rapidly rising trend of PPWR. Understanding the trajectories of PPWR is particularly important, as it is beneficial to discover individuals who are susceptible to excess PPWR and make prevention more targeted. 

There are some strengths of the present study. First, it was a large prospective longitudinal cohort study in a natural context, which tracks the natural course of PPWR. Second, this study first investigated the effects of timing of GWG and pre-pregnancy WC on the trajectories of maternal weight retention in different subpopulations and suggested that late pregnancy is a critical period to control the recommended GWG and reduce higher weight retention. Importantly, using LGMM models, this study can characterize the trajectories of postpartum weight, capture the variability in weight retention or gain over time, and examine the potential risk factors for a different trajectory. Such research is vital to understand the natural course of PPWR, to identify high-risk groups for future maternal overweightness and obesity, and help to design targeted and efficient prevention programs.

There are some limitations to our study. Although the information on postpartum weight mainly relied on measured weight, some of the mother’s postpartum weight was collected by maternal self-report rather than measured weight. However, previous evidence demonstrated that self-reported weight was sufficiently accurate and reliable [36,37]. The sample was rather homogeneous, as participants are older, predominantly from the city, more educated and of a higher social–economic status, representing a healthier segment of the population, which limits the generalizability of the study findings to the general population of childbearing women in China. Our results must also be interpreted cautiously because the participants were only followed up for 8 months, as previous studies have indicated that the first postpartum year was the most key period for natural weight recovery. Additionally, the information on the vomiting/nausea of mothers during the first trimester was not available in this study, which might affect mother’s weight gain in this period. Finally, some factors, such as maternal postpartum diet and levels of physical exercise, were not available in the study, all of which might influence our results. Hence, a well-designed, long-period longitudinal study regarding this topic is needed.

## 5. Conclusions

In summary, three distinct developmental trends of PPWR with significant individual variabilities were identified. Among the obesity-related factors, GWG was the most important predictor for both the baseline weight retention and rates of weight gain or loss. Early targeted interventions should be taken to help women who were multiparous and had a lower pre-pregnancy BMI, higher pre-pregnancy WC, and excessive GWG during late pregnancy, which helps reduce the increasing trend of postpartum weight. 

## Figures and Tables

**Table 1:** Baseline socio-demographic characteristics and the follow-up weight retention of the participants.

Variables	*n*	%
Maternal education level		
Junior or below	33	3.6
High school	112	12.3
College or above	769	84.1
Household income (yuan)		
≤5000	506	54.8
5001–1000	382	41.3
≥10001	36	3.9
Pre-pregnancy WC group (cm)		
<80	760	82.3
≥80	164	17.7
Pre-pregnancy-BMI group (kg/m^2^)		
<18.5	175	18.9
18.5–23.9	609	65.9
24–27.9	108	11.7
≥28	32	3.5
GWG group		
Inadequate	190	20.6
Adequate	429	46.4
Excessive	305	33.0
Parity		
Primiparous	655	70.9
Multiparous	269	29.1
Mode of delivery		
Vaginal delivery or lateral episiotomy	682	73.7
Cesarean delivery	242	26.3
Passive smoke during pregnancy		
No	810	88.7
Yes	104	11.3
Pregnancy-related complications		
No	111	12.0
Yes	813	88.0
Depression at 1 month postpartum		
No	849	91.9
Yes	75	8.1
Exclusive breastfeeding at 1 month postpartum		
No	249	27.0
Yes	672	73.0
Physical exercise at 1 month postpartum		
No	250	27.1
Yes	674	72.9
	**Mean**	**Standard deviation (SD)**
Maternal age (year)	29.90	3.91
Gestational week (week)	38.93	1.51
Waist circumference (cm)	75.64	5.21
Pre-pregnancy BMI (kg/m^2^)	21.25	3.01
Gestational weight gain (kg)	14.37	4.18
PPWR at 1 month postpartum (kg)	5.91	4.78
PPWR at 3 months postpartum (kg)	4.18	4.81
PPWR at 6 months postpartum (kg)	3.25	4.64
PPWR at 8 months postpartum (kg)	2.24	4.66

Abbreviation: BMI, body mass index; GWG, gestational weight gain; PPWR, postpartum weight retention; WC, waist circumference.

**Table 2:** Maternal weight retention for different predictors.

Variables	PPWR at 1 Month, kg			PPWR at 3 Months, kg			PPWR at 6 Months, kg			PPWR at 8 Months, kg		
	Mean	SD	*p*-Value	Mean	SD	*p*-Value	Mean	SD	*p*-Value	Mean	SD	*p*-Value
Maternal education level	F-value = 0.415, *p* = 0.12											
Junior or below	5.55	4.87	0.90	4.05	4.34	0.73	4.58	4.85	0.23	3.64	4.76	0.15
High school	5.95	4.92		3.87	4.61		3.42	4.63		2.60	4.98	
College or above	5.93	4.78		4.25	4.84		3.19	4.63		2.15	4.6	
Household income (yuan)	F-value = 0.485, *p* = 0.62											
≤5000	6.02	4.83	0.29	4.13	4.49	0.88	3.33	4.58	0.84	2.34	4.57	0.80
5001–1000	5.68	4.8		4.21	5.28		3.15	4.76		2.13	4.81	
≥10,001	6.87	4.27		4.52	3.97		3.19	4.26		2.11	4.33	
Pre-pregnancy WC group (cm)	F-value = 7.02, *p* < 0.01											
<80	6.16	4.63	<0.01	4.31	4.52	0.04	3.26	4.5	0.40	2.45	4.50	0.04
≥80	4.76	5.37		3.49	5.87		2.85	5.69		1.52	5.41	
Pre-pregnancy BMI group (kg/m^2^)	F-value = 18.27, *p* < 0.01											
Underweight	7.79	4.22	<0.01	5.6	4.41	<0.01	4.46	4.52	<0.01	3.41	4.36	<0.01
Normal	5.91	4.53		4.1	4.58		3.05	4.44		2.26	4.43	
Overweight	4.31	5.43		3.24	5.38		2.69	5.43		1.46	5.29	
Obesity	1.17	5.31		0.73	6.08		0.2	6.71		-0.73	6.8	
GWG group	F-value = 52.92, *p* < 0.01											
Inadequate	3.04	4.04	<0.01	2.06	4.42	<0.01	1.51	4.51	<0.01	0.85	4.65	<0.01
Adequate	5.37	4		3.9	4.27		3.12	4.25		2.23	4.15	
Excessive	8.47	5.01		5.86	5.12		4.32	5.18		3.24	5.18	
Parity	F-value = 0.59, *p* = 0.44											
Primiparous	6.19	4.77	<0.01	4.09	4.63	0.50	3.19	4.59	0.98	2.36	4.53	0.48
Multiparous	5.20	4.81		4.34	5.19		3.18	5.09		2.10	5.05	
Mode of delivery	F-value = 2.30, *p* = 0.11											
Vaginal delivery or lateral episiotomy	5.35	4.42	0.03	3.91	4.29	0.28	2.78	4.47	0.07	1.77	4.30	0.07
Cesarean delivery	6.14	4.89		4.3	4.95		3.41	4.76		2.40	4.77	
Passive smoke during pregnancy	F-value = 1.04, *p* = 0.31											
No	5.77	4.7	0.03	4.19	4.82	0.89	3.21	4.63	0.44	2.21	4.65	0.67
Yes	6.98	5.28		4.26	4.74		3.64	5.1		2.43	5.07	
Pregnancy-related complications	F-value = 1.41, *p* = 0.24											
No	5.97	4.85	0.32	4.29	4.86	0.06	3.28	4.64	0.62	2.22	4.69	0.68
Yes	5.48	4.38		3.35	4.34		3.04	4.61		2.42	4.41	
Depression at 1 month postpartum	F-value = 0.22, *p* = 0.63											
No	5.88	4.81	0.45	4.21	4.89	0.47	3.29	4.68	0.38	2.29	4.7	0.32
Yes	6.32	4.72		3.8	3.76		2.79	4.13		1.71	4.19	
Exclusive breastfeeding at 1 month postpartum	F-value = 1.18, *p* = 0.31											
No	6.51	4.62	0.02	4.56	4.69	0.13	3.24	4.52	0.90	2.20	4.67	0.99
Yes	5.68	4.85		4.01	4.84		3.20	4.63		2.21	4.61	
Physical exercise at 1 month postpartum	F-value = 0.01, *p* = 0.91											
No	5.83	4.90	0.50	4.27	4.81	0.39	3.10	4.52	0.19	2.16	4.52	0.55
Yes	6.08	4.66		3.95	4.79		3.95	4.81		2.37	4.91	

Abbreviation: BMI, body mass index; GWG, gestational weight gain; PPWR, postpartum weight retention; SD, Standard deviation; WC, waist circumference. F-value was the statistic of the one-way repeated analysis of variance.

**Table 3:** Fit indices for different loading latent growth mixture models for maternal PPWR.

Unconditioned LGMMs	LL value	AIC value	BIC Value	Adjust BIC Value	Entropy	Degrees of Freedom
Linear LGMM	−9510.275	19,044.550	19,102.494	19064.384	0.940	12
Quadratic LGMM	−9458.146	18,950.291	19,032.379	18978.389	0.987	17
Free estimated LGMM	−9515.659	19,059.298	19,126.915	19082.452	0.945	14

Abbreviation: AIC, Akaike Information Criterion; BIC, Bayesian Information Criterion; LL, Likelihood; LGMM, latent growth mixture model; PPWR, postpartum weight retention.

**Table 4:** Fit indices for one- to seven-class growth mixture models for maternal PPWR.

Classes	LL- Value	AIC Value	BIC Value	Adjust BIC Value	LMR-Test	Entropy
2-class	−9458.146	18,950.291	19,032.379	18,978.389	0.006	0.987
**3-class**	**−9430.398**	**18,902.797**	**19,004.199**	**18,937.506**	**0.032**	**0.967**
4-class	−9402.412	18,854.825	18,975.542	18,896.145	0.099	0.904
5-class	−9374.100	18,806.201	18,946.233	18,854.132	0.050	0.897
6-class	−9366.937	18,799.875	18,959.222	18,854.418	0.146	0.900
7-class	−9353.035	18,780.070	18,958.732	18,841.224	0.543	0.900

Abbreviation: AIC, Akaike Information Criterion; BIC, Bayesian Information Criterion; LL, Likelihood; LMR-test, Lo-Mendell-Rubin adjusted likelihood ratio test; PPWR, postpartum weight retention. Bold: More than 90% of participants were classified into Class 3.

**Table 5:** Estimates of covariate prediction of intercept and slopes of the trajectory for PPWR.

Covariates	Intercept		Slope		**Quadratic**	
	β	95% CI	β	**95% CI**	β	**95% CI**
Pre-pregnancy WC	0.11 ***	0.04, 0.19	−0.02	−0.13, 0.09	0.01	−0.06, 0.03
Pre-pregnancy BMI	−0.28 ***	−0.40, −0.16	−0.08	−0.28, 0.12	0.02	−0.03, 0.07
GWG	0.72 ***	0.66, 0.78	−0.26 ***	−0.33, −0.19	0.04 ***	0.02, 0.06
Parity	−0.07	−0.61, 0.48	0.35	−0.34, 1.04	−0.11	−0.28, 0.06
Gestational week	−0.15	−0.32, 0.03	−0.17	−0.47, 0.14	0.05	−0.02, 0.11
Mode of delivery	−0.88 ***	−1.40, −0.35	0.65	−0.08, 1.38	−0.13	−0.31, 0.04
Exclusive breastfeeding at 1 month postpartum	0.72 ***	0.20, 1.23	−0.32	−1.00, 0.36	0.03	−0.13, 0.18

Abbreviation: BMI, body mass index; CI: 95% confidence interval; GWG, gestational weight gain; PPWR, postpartum weight retention; WC, Waist circumference. *** *p* < 0.001.

**Table 6:** Covariates prediction of trajectory class based on the post hoc logistic regressions.

Variables	Class 1		Class 2	
	OR	95% CI	OR	95% CI
Pre-pregnancy WC	1.09	0.97–1.21	0.97	0.91–1.05
Pre-pregnancy BMI	1.28 ***	1.06–1.55	1.41 ***	1.24–1.61
GWG	0.95	0.84–1.07	1.11 **	1.03–1.19
Parity	0.47	0.18–1.22	0.50 *	0.26–0.96
Gestational week	1.38	0.92–2.06	1.26	0.97–1.61
Delivery mode	0.88	0.33–2.38	19.35 ***	2.59–144.51
Exclusive breastfeeding at 1 month postpartum	0.63	0.20–2.02	1.31	0.62–2.75

Abbreviation: WC, Waist circumference; BMI, body mass index; GWG, gestational weight gain; OR, Odds ratio; 95% CI: 95% confidence interval. * *p* < 0.05, ** *p* < 0.01, *** *p* < 0.001.

## Supplementary Materials

The following are available online at https://www.mdpi.com/1660-4601/16/22/4510/s1, Figure S1: The overall trajectory of postpartum weight retention., Table S1: Baseline socio-demographic characteristics and the follow-up weight retention of the participants., Table S2: Estimates of covariate prediction of intercept and slope of the trajectory for PPWR (For early GWG).,Table S3: Estimates of covariate prediction of intercept and slope of the trajectory for PPWR (For late GWG)., Table S4: Estimates of covariate prediction of intercept and slope of the trajectory for PPWR (For very late GWG).
