# Supplementary material for: Patterns of Women’s Postpartum Weight Retention and Its Associations with Maternal Obesity-Related Factors and Parity
Source: Int J Environ Res Public Health. 2019 Nov 15;16(22):4510. doi: 10.3390/ijerph16224510 (PMC6888503; doi:10.3390/ijerph16224510)
Supplement: Supplementary file 1 [file ijerph-16-04510-s001.pdf]

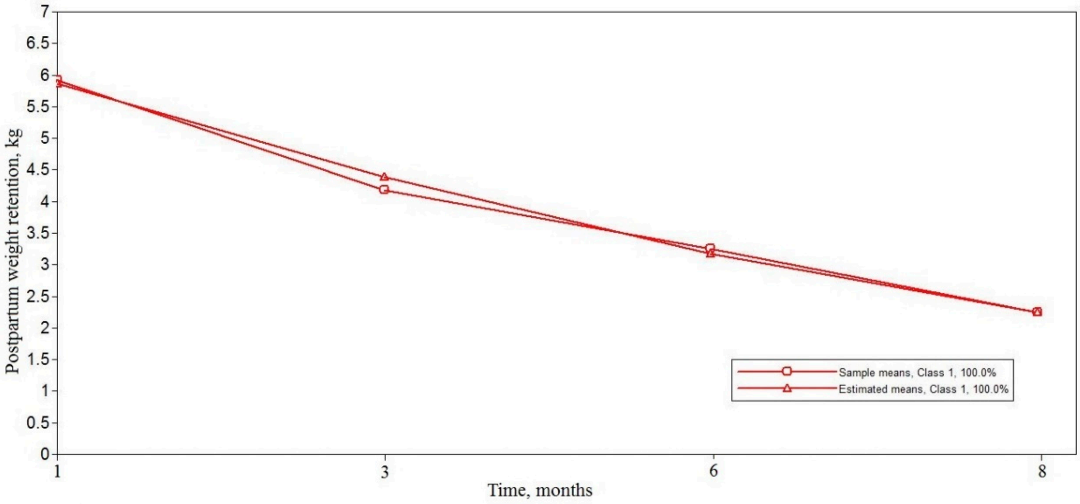

Figure S1. The overall trajectory of postpartum weight retention.

Table S1. Baseline socio-demographic characteristics and the follow-up weight retention of the participants.

Variables	Pre-Pregnancy Waist Circumference (cm)			Pre-Pregnancy-BMI Group (kg/m²)					GWG Group				Parity		
	**< 80**	**≥ 80**	***p***	**<18.5**	**18.5 -23.9**	**24.0-27.9**	**≥28.0**	***p***	**Inadequate**	**Adequate**	**Excessive**	***p***	**1**	**>1**	***p***
Maternal education level															
Junior or below	3.6	3.7	0.97	4.0	3.6	1.9	6.5	0.68	4.3	3.1	3.9	0.64	3.1	4.9	0.41
High school	12.4	11.7		11.6	11.6	16.8	12.9		14.9	11.1	12.2		12.5	11.6	
College or above	84.0	83.6		84.4	84.7	81.3	80.6		80.9	85.8	83.9		84.4	83.6	
Family income (yuan)															
≤5000	54.7	54.9	0.82	57.1	53.4	56.5	62.5	0.42	51.1	54.3	57.7	<0.01	57.4	48.3	0.04
5001-1000	41.2	42.1		37.7	42.7	42.6	31.2		44.2	44.1	35.8		38.9	47.2	
≥10001	4.1	3.0		5.1	3.9	0.9	6.2		4.7	1.6	6.6		3.7	4.5	
Mode of delivery															
Vaginal delivery or lateral episiotomy	72.6	78.5	0.12	62.9	75.5	79.6	78.1	<0.01	67.6	74.9	75.7	0.10	73.4	74.3	0.80
Cesarean delivery	27.4	21.5		37.1	24.5	20.4	21.9		32.1	25.1	24.3		26.6	25. 7	
Passive smoke during pregnancy															
No	89.7	84.2	0.05	91.8	89.1	84.8	78.1	0.08	94.0	90.3	83.2	<0.01	88.9	88.3	0.81
Yes	10.3	15.8		8.2	10.9	15.2	21.9		6.0	9.7	16.8		11.1	11.7	
Pregnancy-related complications															
No	88.8	84.1	0.10	86.9	88.3	87.0	90.6	0.90	85.8	89.0	87.9	0.52	86.4	91.8	0.13
Yes	11.2	15.9		13.1	11.7	13.0	9.1		14.2	11.0	12.1		13.6	8.2	
Depression at 1 month postpartum															
No	91.2	95.1	0.09	88.6	91.8	97.2	93.8	0.08	92.1	90.7	93.4	0.40	90.1	96.3	<0.01
Yes	8.8	4.9		11.4	8.2	2.8	6.2		7.9	9.3	6.6		9.9	3.7	
Exclusive breastfeeding at 1 month postpartum															
No	28.0	22.7	0.30	24.0	29.3	21.5	18.8	0.16	30.0	24.9	28.1	0.37	27.0	27.1	0.96
Yes	72.0	77.3		76.0	70.7	78.5	81.2		70.0	75.1	71.9		73.0	72.9	
Physical exercise at 1 month postpartum															
No	12.8	12.8	0.99	12.1	12.8	13.0	15.6	0.96	10.1.	12.0	15.5	0.17	10.2	19.1	<0.01
Yes	887.2	87.2		87.9	87.2	87.0	84.4		89.9	88.0	84.5		89.8	80.9	

Abbreviation: GWG, gestational weight gain; BMI, body mass index; PPWR, postpartum weight retention.

Table S2. Estimates of covariate prediction of intercept and slope of the trajectory for PPWR (For early GWG).

Covariates	Intercept		Slope		Quadratic	
	β	*p*	***β***	*p*	***β***	*p*
Pre-pregnancy WC	0.098	0.036	-0.056	0.257	0.013	0.296
Pre-pregnancy BMI	-0.617	<0.001	0.100	0.252	-0.009	0.671
Early GWG	0.515	<0.001	-0.159	0.004	0.023	0.080
Parity	-0.567	0.086	0.511	0.142	-0.136	0.142
Gestational week	0.068	0.526	-0.225	0.048	0.054	0.054
Delivery mode	-0.702	0.026	0.425	0.216	-0.102	0.239
Exclusive breastfeeding at 1 month postpartum	0.656	0.034	-0.391	0.239	0.042	0.594

Abbreviation: WC, Waist circumference; BMI, body mass index; GWG, gestational weight gain; PPWR, postpartum weight retention.

Table S3. Estimates of covariate prediction of intercept and slope of the trajectory for PPWR (For late GWG).

Covariates	Intercept		Slope		Quadratic	
	β	*p*	***β***	*p*	***β***	*p*
Pre-pregnancy WC	0.176	<0.001	-0.079	0.216	0.016	0.376
Pre-pregnancy BMI	-0.629	<0.001	0.022	0.784	0.009	0.644
Late GWG	0.518	<0.001	-0.254	<0.001	0.044	<0.001
Parity	-0.202	0.531	0.487	0.133	-0.135	0.162
Gestational week	-0.027	0.815	-0.151	0.242	0.043	0.169
Delivery mode	-1.141	<0.001	0.742	0.090	-0.160	0.126
Exclusive breastfeeding at 1 month postpartum	0.912	0.004	-0.515	0.233	0.066	0.529

Abbreviation: WC, Waist circumference; BMI, body mass index; GWG, gestational weight gain; PPWR, postpartum weight retention.

Table S4. Estimates of covariate prediction of intercept and slope of the trajectory for PPWR (For very late GWG).

Covariates	Intercept		Slope		Quadratic	
	β	*p*	***β***	*p*	***β***	*p*
Pre-pregnancy WC	0.216	0.000	-0.120	0.016	0.023	0.043
Pre-pregnancy BMI	-0.766	0.000	0.071	0.478	0.003	0.900
Early GWG	0.307	0.006	-0.089	0.315	0.010	0.629
Parity	-0.486	0.163	0.677	0.050	-0.178	0.046
Gestational week	0.079	0.490	-0.305	0.034	0.075	0.024
Delivery mode	-1.063	0.002	0.827	0.024	-0.173	0.049
Exclusive breastfeeding at 1 month postpartum	0.097	0.920	-0.086	0.922	0.051	0.790

Abbreviation: WC, Waist circumference; BMI, body mass index; GWG, gestational weight gain; PPWR, postpartum weight retention.
